# Subclinical atherosclerosis and immune activation in young HIV-infected patients with telomere shortening

**DOI:** 10.18632/aging.203350

**Published:** 2021-07-26

**Authors:** María José Alcaraz, Antonia Alcaraz, Raúl Teruel-Montoya, José A. Campillo, Alejandro de la Torre, Ángeles Muñoz, Cristina Tomás, Gabriel Puche, Carlos Báguena, Alfredo Cano, Alfredo Minguela, Enrique Bernal

**Affiliations:** 1 Servicio de Medicina Interna, Sección de Enfermedades infecciosas, Hospital General Universitario Reina Sofía and Universidad de Murcia, Murcia 30003, Spain; 2 Centro Regional de Hemodonación de Murcia, Servicio de Hematología y Oncología Médica, Hospital Universitario Morales Meseguer, IMIB-Arrixaca, Red CIBERER CB15/00055, Murcia 30003, Spain; 3 Servicio de Inmunología, Virgen de la Arrixaca Clinical University Hospital (HCUVA) and Instituto Murciano de Investigación Biosanitaria (IMIB), Murcia 30120, Spain

**Keywords:** young HIV-infected adults, telomere shortening, carotid intima-media thickness, immune activation/senescence

## Abstract

Background: To date, available data on premature aging in young HIV-infected adults are scarce and no reports offer comprehensive assessment of telomere shortening (TS) in relation to subclinical atherosclerosis (SCA). In this study, we investigate if telomere shortening and immune activation markers are associated with SCA, which is one of the main degenerative diseases in young HIV-infected adults.

Methods: A descriptive cross-sectional study was carried out in 149 HIV-infected patients on stable antiretroviral regimen (ART). Carotid intima-media thickness (cIMT) was estimated by carotid ultrasound. Quantitative singleplex PCR was performed to evaluate TS. The expression of activation/senescence markers was evaluated by multiparametric flow cytometry.

Results: TS was observed in 73 patients (49%). Higher cIMT was observed in patients with TS than those without it (0.86 vs. 0.80 mm; p=0.041). Patients under the age of 50 (defined as young adults) with TS showed higher absolute numbers of activated lymphocyte T cells CD8+CD38+ (3.94 vs. 2.34 cell/μl; p=0.07) and lymphocyte B cells CD19+CD38+ (3.07 vs. 2.10 cell/μl; p=0.004) compared to those without TS. In the multivariate analysis, the only factor independently associated with TS was the absolute number of lymphocyte T cells CD8+CD38+ T cells (OR = 1.18; 95%-CI = 1.00-1.39; p = 0.05).

Conclusion: Young HIV-infected adults show premature biological aging with accentuated immune activation. Chronic inflammation with excessive T-cells activation could be associated to TS, premature aging, and SCA in young HIV-infected adults.

## INTRODUCTION

Telomeres are regions at each end of chromosomes which protects them from deterioration or from fusion with neighboring chromosomes [1]. Mammalian telomeres are composed of very long arrays (2–100 kb) of TTAGGG repeats that are maintained by telomerase. Telomere shortening (TS) largely reflects the replicative histories of stem cells and progenitor cells on top of the somatic cells [2]. Without the presence of telomeres, the 3-'OH end of the DNA would be unprotected and would be susceptible to being damaged during cell replication. With aging, these telomeres are shortened until the genetic material is exposed, causing cellular senescence associated with a dangerous loss of DNA [3].

Shortening of the leukocyte telomere length is observed long before the onset of carotid lesions and can predicts its progression. Besides, it is associated with cardiovascular disease (CVD) and the severity of atherosclerotic plaques in coronary and carotid arteries [4]. Carotid intima-media thickness (cIMT) has been used to estimate atherosclerotic damage. cIMT is a valid measurement of subclinical atherosclerosis (SCA), since it has been consistently related to future cardiovascular events in population studies [5]. cIMIT also correlates with the extension of coronary atherosclerosis [5, 6].

Chronic inflammation can accelerate telomere dysfunction and cell senescence in HIV-infected population [7]. With aging, cardiovascular diseases in HIV-infected patients become especially problematic. Emerging data indicate that, even under a strict control of the traditional cardiovascular risk factors, HIV-infection increases rates of atherosclerosis-related disease, mostly due to chronic arterial inflammation and the injury induced by chronic immune response, which, in turn, promotes dysfunction of the endothelium, atherosclerosis [8], and thrombosis. In fact, endothelial injury and dysfunction have been proposed as plausible links between HIV infection [9] and atherosclerosis. Besides, despite effective viral suppression, immune activation leads to premature onset of immune-senescence, which could be related to the earlier aging of HIV-infected patients [8].

Several immunological biomarkers have been associated with the immune senescence related with aging, such as TS, the accumulation of CD28 negative T-cells (CD28^null^), and the increase in the expression of activation markers, i.e. CD38, HLA-DR [10]. Chronic viral infections like HIV are considered major contributors to immune senescence and chronic inflammatory state observed in elderly HIV-infected patients [11].

To date, available data on premature aging in young HIV-infected adults are limited [12] and no reports offer a comprehensive assessment of TS, a key molecular marker of biological aging, in relation to SCA and/or activation/senescent profiles. In this paper, we investigate if telomere shortening and immune activation markers are associated with SCA, which is one of the main degenerative diseases observed in young HIV-infected adults.

## MATERIALS AND METHODS

### Study design, participants, setting, and eligibility

This descriptive cross-sectional study was performed in a sample of 149 HIV-infected patients on stable antiretroviral therapy (ART) in Reina Sofía University Hospital in Murcia, Spain. 91% of our patients had undetectable HIV viral load during the study. Patients over the age of 18 were recruited if documented HIV infection. The average time of our patients with HIV infection was 13 years. Concomitant pathologies or treatments were not exclusion criteria. The study conformed principles of the Declaration of Helsinki and the Good Clinical Practice Guidelines, and was approved by the local ethics committee (“Comité Etico de Investigación Clínica del Hospital Universitario Reina Sofía de Murcia”). All patients gave their written consent to participate in the study [13].

Medical records were carefully reviewed, and all patients underwent a physical examination. Gender, age, body mass index, smoking status, family history of CVD, and treatments with antiretroviral drugs were recorded. The presence of arterial hypertension, hypercholesterolemia, and hypertriglyceridemia was defined according to the Adult Treatment Panel III criteria. A sample of fasting venous blood was obtained to determine concentrations of glucose, high-sensitivity C-reactive protein (hsCRP), creatinine, total cholesterol, D-dimer, HDL cholesterol, and triglycerides using standard enzymatic methods. Concentration of LDL cholesterol was calculated using the Friedewald equation [14]. Plasma viral load was measured using the Cobas TaqMan HIV-1 assay (RocheDiagnostics Systems, Branchburg, NJ, USA). CD4 and CD8 T-cell counts were determined by flow cytometry (Becton Dickinson, NJ, USA) [14].

### cIMT measurement

Carotid measurement was performed during the baseline visit. For the determination of carotid intima-media thickness (cIMT), B-mode high-resolution ultrasound was used following a standard procedure previously described [12, 13]. All measurements were performed by the same researcher, who was unaware of the group to which the patients belonged. SCA was considered if IMT was higher than 0.8 mm in common carotid, higher than 1.0 mm in bulb carotid, or there was a plaque in the carotid artery [13, 14].

### Telomere length measurement in blood leukocyte

Genomic DNA was extracted from whole venous blood following standard procedures [15] and stored at 4° C in TE buffer (10mM Tris–HCl, 0.1mM EDTA, pH7.5) at a concentration of ~ 100 ng/ml. DNA stocks were diluted with pure water just prior to singleplex quantitative polymerase chain reaction (qPCR) [16]. Mean relative telomere length (TL) was then assayed with a monochromatic multiplex qPCR (MMqPCR) assay developed by Cawthon [17]. Primers for the single copy gene albumin - albu (5' CGG CGG CGG GCG GCG CGG GCT GGG CGG AAA TGC TGC ACA GAA TCC TTG 3', and albd (5' GCC CGG CCC GCC GCG CCC GTC CCG CCG GAA AAG CAT GGT CGC CTG TT 3')- and for telomere - telg (5' ACACTA AGG TTT GGG TTT GGG TTT GGG TTT GGG TTA GTG T 3'), and telc (5' TGT TAG GTA TCC CTA TCC CTA TCC CTA TCC CTA TCC CTA ACA 3')- were used at 0.9 μM final concentration. The thermal cycling profile was 95° C for 15min, followed by 2 cycles of 94° C for 15s, 49° C for 15s, followed by 40 cycles of 94° C for 15s, 62° C for 10s, 74° C for 15s, 84° C for 10s, and 88° C for 15s, with signal acquisition at the end of 74° C or 88° C steps, for telomere or albumin reaction, respectively [17]. Reactions were carried out in triplicate in a 10μL volume using the SYBR Select Master Mix (Applied Biosystems by Life Technologies) on a LightCycler 480 (Roche). A standard curve prepared with human blood DNA was included in each run and used to estimate telomere (T) and single nuclear gene (S) [18].

The whole blood DNA concentrations were confirmed to lie well within the linear range of the standard curves. Relative TL was expressed as the average T/S ratio of triplicates [17]. As validation controls of the technique, we use DNA from the elderly with age > 88.

TS was considered if the value of TL was less than 200. We established 200 as a cut-off point, since it was the median value obtained in our sample.

### Flow cytometry

EDTA anticoagulated peripheral blood cells were labeled following a lyse/wash protocol with an 8-color/9-monoclonal antibody (mAb). In this study the strategy previously described by Bernal et al. was used to label, acquire and analyze the blood cells in flow cytometry [19].

The expression of CD28, CD38, CD86, and HLA-DR activation/senescence markers were evaluated as percentage (%) and absolute numbers (cells/μl) of positive cells as well as mean fluorescence intensity (MFI) of the marker on CD3+CD4+ and CD3+CD8+ T lymphocytes, CD19+ B lymphocytes, CD3-CD19-CD16+ NK lymphocytes, CD4+CD86+HLA-DR+ medium SSC monocytes, CD16++ elevated SSC granulocytes, and elevated SSC auto fluorescent eosinophils [19].

### Statistical analyses

A descriptive analysis of patients’ characteristics was conducted using frequency tables for categorical variables and mean and SD for continuous variables. Differences in categorical variables between patients with and without TS were assessed through the X2 test or the Fisher test, and t student tests for continuous variables. Binary logistic regression was used to evaluate the independent variable association with TS. Multivariable models were adjusted for age, transmission group (homosexual/bisexual, injecting drug use, heterosexual, and other/unknown), Framingham risk score, and variables that were significant in the univariate analysis. Wald tests were used to derive P values. Significance was placed at P<0.05. All statistical analyses were performed using SPSS package version 24 [13]. The variables collinearity were analyzed, and those that were collinear were not included.

## RESULTS

Detailed biological and clinical characteristics of patients at the inclusion of the study are shown in Table 1. In summary, the study comprised 149 HIV-infected patients whose mean age was of 48.57±10.15 years, 78.5% men, 91% with HIV viral load lower than 20 copies/mL for longer than 12 months, and an average CD4+ T cell count of 736 cells/mL. HIV-infected subjects were on stable ART, 26.8% (n=40) on protease inhibitor (PI)-based regimen, 32% (n=47) on Non-nucleoside reverse transcriptase inhibitors (NNRTIs)-based regimen, and 51% (n=75) on integrase inhibitor (IIS)-based regimen in combination with Nucleoside Analogue Reverse Transcriptase Inhibitor (NRTI). Main CVD risk factors were hypertension (n=27, 18.2 %), type 2 diabetes (n=8, 5.4%), dyslipidemia (n=47, 31.5%), current smoking habit (n=81, 57.6%), and carotid SCA (n=61, 44.2%).

**Table 1:** Basal biological and clinical characteristics of HIV-infected patients.

**Variable**	**n = 149**
Age, yrs [mean (SD)]	48.57 (10.15)
Sex [male, (%)]	117 (78.5)
Transmission group, (%)	
*Homosexual/bisexual*	42.6
*Heterosexual*	37.2
*Intravenous drug users*	25
*Others/unknown*	3.4
Type 2 diabetes, n (%)	8 (5.4)
Hypertension, n (%)	27 (18.2)
Dyslipidemia, n (%)	47 (31.5)
Current Smoker, n (%)	81 (55,5)
ART, years [mean (SD)]	4.1 [2.0, 9.0]
Alcohol consumption, n (%)	74 (51.0)
Drug user, n (%)	19 (13.4)
Coronary heart disease, n (%)	7 (4.8)
Stroke, n (%)	2 (1.4)
AIDS (%)	31 (23.0)
On ART, n (%)	
*Protease inhibitor, n (%)*	40 (26.8)
*NNRTI, n (%)*	47 (32.0)
*Integrase inhibitor, n (%)*	75 (51.0)
*Other ART combination (%)*	23 (15.6)
Carotid plaques, n (%)	23 (16.7)
Carotid SCA, n (%)	61 (44.2)
CD4 nadir	256.00 [119.50, 422.50]
BMI	26.47 [23.59, 28.84]
CD4+ T-cell count, cells/mL	736.00 [471.00, 966.50]
CD4/CD8 ratio	0.83 [0.51, 1.10]
Left common carotid IMT	0.66 [0.56, 0.76]
Framingham risk score	3.00 [1.82, 4.25]
Fold TS	207.94 [152.22, 286.03]

AIDS, acquired immune deficiency syndrome; ART, antiretroviral therapy; BMI, body mass index; NNRTI, non-nucleoside reverse transcriptase inhibitor; SCA, subclinical atherosclerosis; IMT, intima–media thickness; TS, telomere shortening.

TS was observed in 73 (49%) patients (Table 2). Compared to patients without TS, those with TS were older (50±10 vs. 46±10 years; p=0.01); had higher cIMT in the left carotid bulb (0.86 [0.74-1.03] vs. 0.80 [0.72-0.94] mm; p = 0.041), left common carotid artery (0.70 [0.56-0.80] vs. 0.63 [0.52 - 0.71] mm; p = 0.01), and right common carotid artery (0.68 [0.58-0.79] vs. 0.60 [0.53-0.70] mm; p = 0.018); are more likely to have carotid plaque (23.9% vs. 9.9%; p = 0.048), and had a higher score on the Framingham scale (4 [2-5] vs. 2 [1- 4]; p = 0.011). Additional clinical and HIV-related parameters stratified by TS are shown in (Table 2).

**Table 2:** Clinical and HIV-related parameters stratified by telomere shortening (TS).

	**No ST (N=76)**	**ST (N=73)**	**P**
Age, yrs [mean (SD)]	46.49 (9.94)	50.74 (9.97)	0,010
Sex [male (%)]	58 (76.3)	59 (80.8)	0,638
Transmission group, (%)			0,334
Homosexual/bisexual	35 (46.1)	28 (38.9)	
Heterosexual	24 (31.6)	31 (43.1)	
Intravenous drug users	13 (17.1)	12 (16.7)	
Others/unknown	4 (5.3)	1 (1.4)	
Type 2 diabetes, n (%)	1 (1.3)	9 (12.5)	0,017
Hypertension, n (%)	7 (9.2)	20 (27.8)	0,007
Dyslipidemia, n (%)	23 (30.3)	24 (32.9)	0,867
Smoker, n (%)			0,794
Nonsmoker	32 (42.1)	30 (42.9)	
Current smoker	43 (56.6)	38 (54.3)	
Past smoker	1 (1.3)	2 (2.9)	
Drug user, n (%)	12 (16.4)	7 (10.1)	0,393
Exercise, n (%)			0,809
No exercise	21 (29.6)	20 (30.3)	
Mild	10 (14.1)	13 (19.7)	
Moderate	17 (23.9)	15 (22.7)	
Intensive	23 (32.4)	18 (27.3)	
Coronary heart disease, n (%)	3 (4.1)	4 (5.6)	0,955
Carotid plaques, n (%)	7 (9.9)	16 (23.9)	0,048
Carotid SCA, n (%)	27 (38.0)	34 (50.7)	0,183
Left carotid bulb IMT mm*	0.80 [0.72-0.94]	0.86 [0.74, 1.03]	0,041
Left common carotid IMT*	0.63 [0.52-0.71]	0.70 [0.56, 0.80]	0,01
Framingham risk score*	2.00 [1.00-4.00]	4.00 [2.00-5.00]	0,011
Stroke, n (%)	0 (0.0)	2 (2.9)	0,446
Hepatitis C virus Ab, n (%)	15 (20.5)	15 (21.4)	1,0
SVS, n (%)			0,560
No SVS	1 (8.3)	1 (9.1)	
Yes SVS	11 (91.7)	9 (81.8)	
Others/unknown	0 (0.0)	1 (9.1)	
HBSAg (%)	1 (8.3)	0 (0.0)	1,0
CD4 nadir*	249.0 [141.0, 367.2]	268.0 [119.0, 443.0]	0,615
CDC HIV stage, n (%)			0,194
Stage A	32 (48.5)	21 (35.6)	
Stage B	23 (34.8)	21 (35.6)	
Stage C	11 (16.7)	17 (28.8)	
AIDS (%)	11 (16.2)	20 (29.9)	0,092
On ART, n (%)			0,566
Protease inhibitor, n (%)	20 (26.3)	20 (27.4)	1,0
NNRTI, n (%)	25 (32.9)	22 (31.0)	0,943
Integrase inhibitor, n (%)	35 (46.1)	40 (56.3)	0,279
Other ART combination (%)	12 (15.8)	11 (15.5)	1,0
Time on ART, yrs [mean (SD)]	3.83 [1.91, 6.69]	4.38 [2.14, 9.67]	0,461
BMI*	25.55 [23.52-28.47]	27.19 [23.84-29.84]	0,173
Glomerular filtration mL/min*	89.2 [84.29-94.20]	84.6 [74.27-95.10]	0,199
Glucose level-mg/dL*	91.0 [85.50-99.00]	95.0 [86.00-106.00]	0,122
Total cholesterol level-mg/dL*	182.0 [157.50-221.00]	184.0 [159.00-216.00]	0,905
LDL cholesterol level-mg/dL*	108.0 [85.00-131.50]	108.0 [86.50-133.00]	0,908
HDL cholesterol level-mg/dL*	122.0 [87.00-171.00]	124.0 [87.00-195.00]	0,869
Triglyceride levels-mg/dL*	45.0 [38.00-55.00]	47.0 [41.00-54.00]	0,621
CD4+ T-cell count-cells/mL*	769.0 [531.0-980.2]	709.0 [466.0-919.0]	0,179
CD4/CD8 ratio*	0.77 [0.56-1.1]	0.86 [0.44-1.17]	0,848
Fold TS*	284.1 [243.8-383.3]	151.2 [108.4-171.2]	0,001

*median [IQR].

SVS, Sustained Viral Suppression; HBSAg, hepatitis B surface antigen; CDC, Centers for Disease Control and Prevention; AIDS, acquired immune deficiency syndrome; ART, antiretroviral therapy; BMI, body mass index; HDL, high-density lipoproteins; LDL, low-density lipoproteins; NNRTI, nonnucleoside reverse transcriptase inhibitor; SCA, subclinical atherosclerosis.

A multivariate analysis including significant variables in the univariate analysis showed that the only factor associated with TS was the cIMT of the left carotid artery (OR=9.1, 95%-CI=1.10-76; p=0.04). A weak and inverse correlation was found between TS and left carotid cIMT (rho=-0.201; p=0.018) and right carotid cIMT (rho=-0.203; p=0.017).

**Figure 1:**
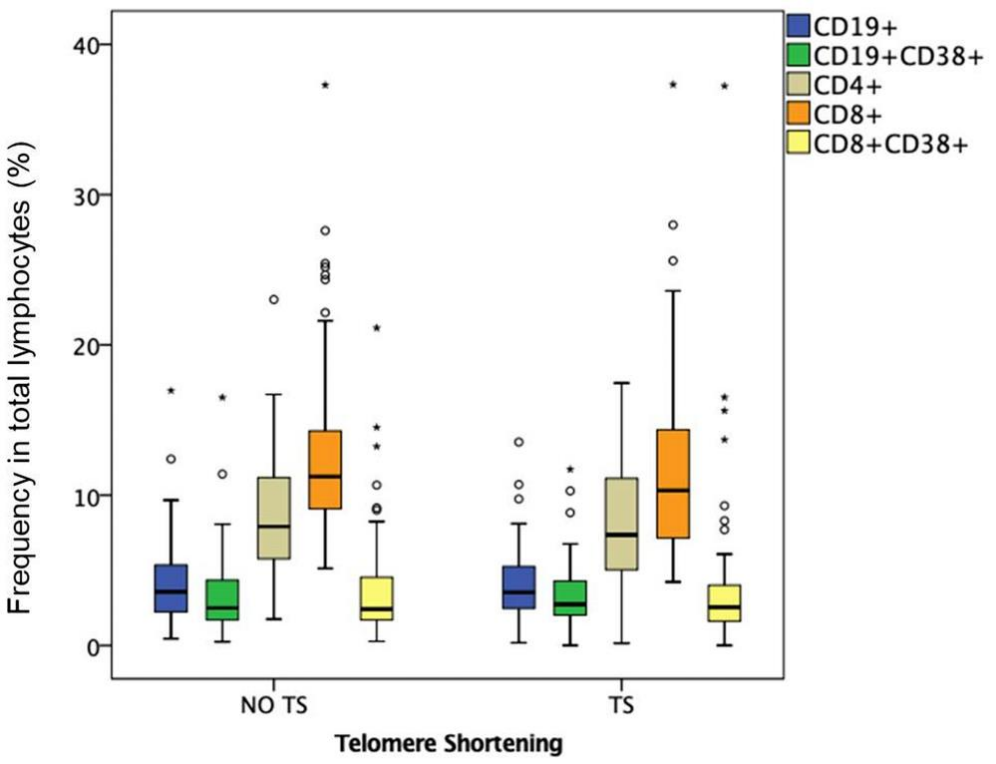
**Frequency in total lymphocytes (%) in HIV-infected young adults with and without TS.** Box-plots show the percentage of total and activated B and T lymphocytes: CD19+ B lymphocytes, CD19+ CD38+ activated B lymphocytes, CD4+ helper T lymphocytes, CD8+ cytotoxic T lymphocytes, CD8+ CD38+ activated cytotoxic T lymphocytes.

**Figure 2:**
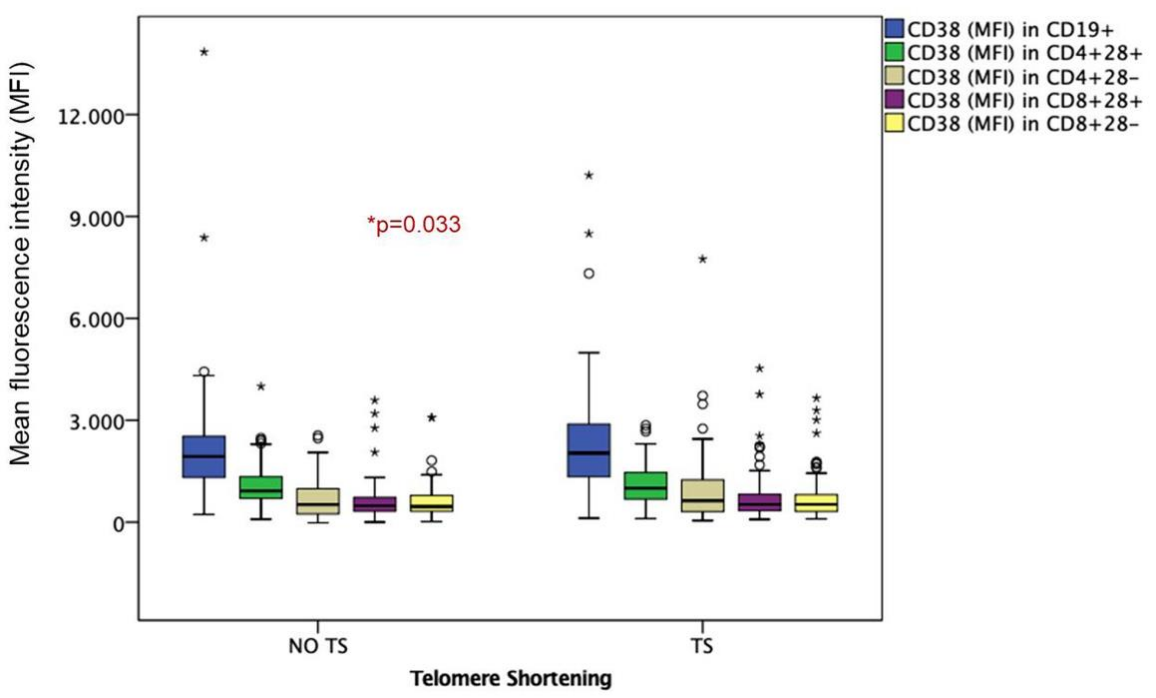
**Mean fluorescence intensity (MFI) in HIV-infected young adults with and without TS.** Box-plots show MFI of senescent and non-senescent activated B and T lymphocytes: CD38 in CD19+ activated B lymphocytes, CD38 in CD4+28+ Non-senescent activated helper T lymphocytes, CD38 in CD4+28- senescent activated helper T lymphocytes, CD38 in CD8+28+ Non-senescent activated cytotoxic T lymphocytes, CD38 in CD8+28- senescent activated cytotoxic T lymphocytes.

For a better understanding of the role of immunological variables in the TS, subsequent analyses were carried out in patients under the age of 50 (young adults, 69 out of 149). TS was observed in 45% (n=31 out of 69) of young HIV-infected adults (Table 3). Compared to patients without TS, those with TS were older (43±5 vs. 39±7 years; p=0.01) and had higher levels of absolute counts of activated CD8+CD38+ T cells (3.94 [2.46-5.51 cell/μl] vs. 2.34 [1.68-4.55 cell/μl]; p=0.07), activated CD19+CD38 B cells (3.07 [2.46-3.99 cell/μl] vs. 2.10 [1.59 -2.98 cell/μl]; p=0.004), activated/senescent CD4+CD28-CD38+ T cells (870.0 [466.5-1472.0 cell/μl] vs. 405.0 [242.7-912.5 cell/μl]; p=0.03) and activated CD8+CD38+ T cells (609.0 [475.5-1212.5 cell/μl] vs. 508.5 [328-861.25 cell/μl]; p=0.05) (Figures 1, 2). In the multivariate analysis, independent variables associated with TS were age (OR=1.14; 95%-CI=1.04-1.25; p=0.04) and absolute counts of activated CD8+CD38+ T cells (OR=1.18; 95%-CI=1.00-1.39; p=0.05).

**Table 3:** Clinical and HIV-related parameters stratified by telomere shortening (TS) in patients <50 years.

	**No ST (N=38)**	**ST (N=31)**	**P**
Age, yrs [mean (SD)]	38.82 (6.86)	42.77 (5.16)	0.010
Sex [male (%)]	32 (84.2)	27 (87.1)	1.00
Transmission group, (%)			0.371
Homosexual/bisexual	24 (63.2)	17 (54.8)	
Heterosexual	8 (21.1)	11 (35.5)	
Intravenous drug users	4 (10.5)	3 (9.7)	
Others/unknown	2 (5.3)	0 (0.0)	
Type 2 diabetes, n (%)	0 (0.0)	2 (6.5)	0.386
Hypertension, n (%)	2 (5.3)	3 (9.7)	0.813
Dyslipidemia, n (%)	10 (26.3)	6 (19.4)	0.693
Smoker, n (%)			0.712
Nonsmoker	18 (47.4)	13 (43.3)	
Current smoker	19 (50.0)	15 (50.0)	
Past smoker	1 (2.6)	2 (6.7)	
Drug user, n (%)	6 (16.7)	2 (6.7)	0.389
Exercise, n (%)			0.992
No exercise	12 (34.3)	11 (37.9)	
Mild	5 (14.3)	4 (13.8)	
Moderate	8 (22.9)	6 (20.7)	
Intensive	10 (28.6)	8 (27.6)	
Coronary heart disease, n (%)			
Carotid plaques, n (%)	0 (0.0)	2 (6.9)	0.392
Carotid SCA, n (%)	8 (22.9)	9 (31.0)	0.651
Left carotid bulb IMT mm*	0.77 [0.63, 0.87]	0.86 [0.73, 0.90]	0.071
Left common carotid IMT*	0.56 [0.50, 0.64]	0.60 [0.53, 0.70]	0.048
Framingham risk score*	1.00 [1.00, 2.00]	2.00 [1.00, 3.00]	0.052
Stroke, n (%)	1 (2.6)	0 (0.0)	1.00
Hepatitis C virus Ab, n (%)	2 (5.6)	4 (12.9)	0.534
SVS, n (%)			
No SVS			
Yes SVS			
Others/unknown			
HBSAg (%)	1 (11.1)	0 (0.0)	1.00
CD4 nadir*	290.00 [169.00, 448.00]	256.00 [54.50, 381.50]	0.164
CDC HIV stage, n (%)			0.166
Stage A	21 (60.0)	11 (37.9)	
Stage B	8 (22.9)	8 (27.6)	
Stage C	6 (17.1)	10 (34.5)	
AIDS (%)	6 (15.8)	11 (35.5)	0.108
On ART, n (%)			0.874
Protease inhibitor, n (%)	6 (15.8)	8 (25.8)	0.466
NNRTI, n (%)	16 (42.1)	10 (32.3)	0.555
Integrase inhibitor, n (%)	18 (47.4)	16 (51.6)	0.913
Other ART combination (%)	5 (13.2)	4 (13.3)	1.00
Time on ART, yrs [mean (SD)]	3.17 [1.79, 4.99]	2.80 [1.69, 6.97]	0.800
BMI*	26.48 [23.61, 28.56]	25.83 [23.09, 28.11]	0.708
Glomerular filtration mL/min*	89.95 [85.79, 95.66]	92.03 [75.22, 101.96]	0.818
Glucose level-mg/dL*	90.00 [84.00, 97.00]	90.00 [85.00, 99.50]	0.575
Total cholesterol level-mg/dL*	176.00 [157.00, 203.00]	172.00 [150.50, 211.50]	0.995
LDL cholesterol level-mg/dL*	104.00 [80.00, 120.00]	105.00 [82.00, 136.00]	0.821
HDL cholesterol level-mg/dL*	44.00 [38.00, 53.00]	47.00 [41.00, 53.50]	0.839
Triglyceride levels-mg/dL*	119.00 [86.00, 148.00]	104.00 [84.00, 163.50]	0.749
CD4+ T-cell count-cells/mL*	827.0 [434.00, 967.00]	683.0 [435.00, 931.00]	0.431
CD4/CD8 ratio*	0.80 [0.51, 1.09]	0.80 [0.42, 1.03]	0.743
Fold TS*	286.03 [247.28, 389.38]	150.12 [105.80, 170.08]	<0.001

*median [IQR].

SVS, Sustained Viral Suppression; HBSAg, hepatitis B surface antigen; CDC, Centers for Disease Control and Prevention; AIDS, acquired immune deficiency syndrome; ART, antiretroviral therapy; BMI, body mass index; HDL, high-density lipoproteins; LDL, low-density lipoproteins; NNRTI, nonnucleoside reverse transcriptase inhibitor; SCA, subclinical atherosclerosis.

## DISCUSSION

Although ART drastically reduces immune activation, HIV-1-infected individuals show signs of persistent immune activation and inflammation [20]. In fact, HIV-1 infected patients on ART with suppressed viraemia have higher rates of age-associated diseases and shorter life expectancy, compared to uninfected persons of the same age [21, 22]. Our study reveals that in young adults immune-activation is related to telomere shortening, and all together could contribute to the increased co-morbidity described in HIV disease. As previously described in adults and older children [23, 24], HIV infection is associated with increased levels of activated CD8+ effector T cells, which lead to the accumulation of cells with a senescent phenotype (CD28-). It is well known that a major driver of cellular senescence is the telomere shortening [25]. These data are consistent with a previous report showing that high percentage of CD8+CD28- correlates with shorter telomeres [26].

Data described in this manuscript suggests that young HIV-infected adults accumulate activated CD8+CD38+ T cells together with senescent T cells (CD28-). The finding that activated and depleted CD8 cells are negatively correlated with telomere length supports the view that sustained immune activation and cell depletion are closely related to accelerated biological aging and increased comorbidities in young HIV-infected patients. In fact, in our study, telomere length is inversely associated with increased cIMT and, therefore, with subclinical atherosclerosis.

Although it has been described that some ARTs such as those using nucleoside-like reverse transcriptase inhibitors are linked to a more pronounced telomere shortening, possibly by inducing the inhibition of the telomerase enzyme responsible for maintaining telomere length [20], our results could not detect any association between the type of ART and the telomere length. However, importantly, it has been clearly demonstrated that shortened telomeres are associated to excessive cellular replication occurring as a consequence of the chronic immune activation triggered by latent HIV infection [20].

In line with systematic reviews and meta-analysis describing that HIV-infected individuals have significantly higher values of cIMT than uninfected ones [27], our data show that forty nine percent of patients with TS had increased cIMT in the left carotid bulb, the left common carotid artery, and the right common carotid artery, in addition to higher probability of carotid plaque and a higher score on the Framingham scale than those without TS. In fact, in our study, TS was an independent predictor of SCA, measured by cIMT, in young HIV-infected patients in stable ART. These results are the basis to explain the higher prevalence of atherosclerosis associated to the chronic inflammation induced by HIV infection and the use of ART, and make up the best scenario to boost TS.

We used qPCR to assay the telomere length, as it is supposed to be quicker, more sensitive, and less technical than other methods, so it allows for a higher throughput [28]. This higher throughput also serves to reduce the costs [28]. Moreover, the qPCR method is associated with less stringent requirements on DNA amount and quality. However, the qPCR only determines the relative mean telomere length. Furthermore, the data obtained using this technique are not presented in absolute values of kilobase pairs (kbp) [28].

Shortened telomeres in vascular endothelium are thought to promote cellular senescence, which feeds the inflammatory cycle, leading to plaque deposition [29, 30, 8]. Thus, higher level of inflammation could cause endothelial dysfunction, which is seen as the link between infection and atherosclerosis [31, 32]. The internal mammary artery has longer telomeres than other arteries and is protected from atherosclerosis. Therefore, increased telomerase activity protects endothelium from senescence, suggesting that telomeres play a protective role in atherosclerosis. In this line, it has been shown that shortened telomeres in ageing cardiomyocytes leads to cell loss by increased cellular senescence and apoptosis, and limits proliferative potential of cardiac progenitors, contributing to heart failure [29, 30, 33, 8, 10].

Altogether, our data support a scenario in which viremia sustained chronic inflammation drives to a high turnover of naïve immune cells, which rapidly differentiate and get exhausted, resulting in the accumulation of senescent cells with shortened telomeres. Senescent cells, in turn, secrete pro-inflammatory factors, which would reinforce inflammation creating a positive feedback loop. As expected, in our series, HIV-infected patients with TS had higher levels of activation markers (CD38 expression) on T and B cells, which agree with other studies describing the association of increased immune activation (higher percentage of CD4+CD38+ T cells) with shorter telomeres in HIV-1-infected patients on ART. In line with previous studies [34], our data suggest that chronic inflammation associated with HIV-1 infection drives to an excessive activation and proliferation of T cells, which in turn leads to telomere shortening and ultimately to immunosenescence. As a consequence, young HIV-infected adults with TS would be at higher risk of presenting comorbidities and non-AIDS events.

Our study has some limitations that must be taken into account, for example, its cross-sectional nature that means that the relationships found can not be classified as causal. On the other hand, there are factors that were not evaluated in this study, such as oxidative stress, which are more frequent in HIV infected patients and have been associated with persistent chronic inflammation, early aging and greater telomere shortening [35, 36]. Nor have we evaluated antioxidant treatment. However, we observed that there is an association between cell activation markers with telomeric shortening, which could help to better understand the early aging of this population.

In conclusion, young HIV-infected adults exhibit premature biological ageing with higher SCA, shortened telomeres, and increased T-cell immune activation and senescence, compromising their immune surveillance and increasing the risk of age-related diseases and non-AIDS events. Nonetheless, new studies will be necessary to investigate the mechanisms involved, which could reveal potential targets to improve the quality of life and survival of these patients.
